# Long-term incidence and prognostic factors of the progression of new coronary lesions in Japanese coronary artery disease patients after percutaneous coronary intervention

**DOI:** 10.1007/s00380-013-0382-6

**Published:** 2013-06-27

**Authors:** Hidehiro Kaneko, Junji Yajima, Yuji Oikawa, Shingo Tanaka, Daisuke Fukamachi, Shinya Suzuki, Koichi Sagara, Takayuki Otsuka, Shunsuke Matsuno, Hiroto Kano, Tokuhisa Uejima, Akira Koike, Kazuyuki Nagashima, Hajime Kirigaya, Hitoshi Sawada, Tadanori Aizawa, Takeshi Yamashita

**Affiliations:** The Department of Cardiovascular Medicine, The Cardiovascular Institute, 3-2-19 Nishiazabu, Minato-ku, Tokyo, 106-0031 Japan

**Keywords:** New culprit coronary artery lesion, Percutaneous coronary intervention, High-density lipoprotein cholesterol, Insulin, Japanese

## Abstract

Revascularization of an initially non-target site due to its progression as a new culprit lesion has emerged as a new therapeutic target of coronary artery disease (CAD) in the era of drug-eluting stents. Using the Shinken database, a single-hospital-based cohort, we aimed to clarify the incidence and prognostic factors for progression of previously non-significant coronary portions after prior percutaneous coronary intervention (PCI) in Japanese CAD patients. We selected from the Shinken database a single-hospital-based cohort of Japanese patients (*n* = 15227) who visited the Cardiovascular Institute between 2004 and 2010 to undergo PCI. This study included 1,214 patients (median follow-up period, 1,032 ± 704 days). Additional clinically driven PCI to treat previously non-significant lesions was performed in 152 patients. The cumulative rate of new-lesion PCI was 9.5 % at 1 year, 14.4 % at 3 years, and 17.6 % at 5 years. There was no difference in background clinical characteristics between patients with and without additional PCI. Prevalence of multi-vessel disease (MVD) (82 vs. 57 %, *p* < 0.001) and obesity (47 vs. 38 %, *p* = 0.028) were significantly higher and high-density lipoprotein cholesterol (HDL) level (51 ± 15 vs. 47 ± 12 mg/dl, *p* < 0.001) was significantly lower in patients with additional PCI than those without. Patients using insulin (6 vs. 3 %, *p* = 0.035) were more common in patients with additional PCI. Multivariate analysis showed that MVD, lower HDL, and insulin use were independent determinants of progression of new culprit coronary lesions. In conclusion, progression of new coronary lesions was common and new-lesion PCI continued to occur beyond 1 year after PCI without attenuation of their annual incidences up to 5 years. Greater coronary artery disease burden, low HDL, and insulin-dependent DM were independent predictors of progression of new culprit coronary lesions.

## Introduction

Drug-eluting stents have dramatically decreased stent restenosis and target lesion revascularization [1]. Previous clinical studies validated the pathogenesis and risk factors for stent restenosis, suggesting that restenosis rates differed in patients’ clinical and angiographic characteristics such as diabetes mellitus, vessel size, lesion length, and repeated revascularization procedures after stent implantation [2–4].

This has provided us next unresolved problems regarding revascularization of initially non-target site due to its progression as a new culprit lesion after percutaneous coronary intervention (PCI). However, the clinical evidence about the long-term incidence and risk factors of the development of new lesions after PCI was limited.

We conducted a study of a hospital-based cohort from the Shinken database using data during the period between 2004 and 2010 [5]. Among the cohort identified, the patients who underwent PCI (*n* = 1214) were enrolled in the present study and evaluated prognostic factors for the progression of new coronary lesion in Japanese CAD patients after PCI.

## Methods

### Study population and study protocol

The Shinken database was established comprising all the new patients visiting the Cardiovascular Institute Hospital in Tokyo, Japan (“Shinken” is an abbreviated name in Japanese for the name of the hospital), and excluded patients with active cancer as well as any foreign travelers. The principle aim of this hospital-based database is a surveillance of the prevalence and prognosis of cardiovascular diseases in the urban areas of Japan [6]. The registry started in June 2004, and thereafter patients have been continually registered to the database annually. The data in the present study was derived from this database between June 2004 and March 2011 (Shinken database 2004–2010) including 15,227 new visiting patients. Among them, patients who underwent PCI at our institute (*n* = 1,214) were enrolled in the present study.

### Patient follow-up

The health status and the incidence of cardiovascular events and mortality are maintained in the database by being linked to the medical records of the hospital, and by study documents of prognosis sent once per year to those who stopped hospital visits or who were referred to other hospitals.

In the present data analysis, the follow-up data after April 1, 2011, were excluded. Therefore, the end of the follow-up period was defined as one of the following three; (1) the data of death, if the date was prior to March 31, 2011: (2) the final hospital visit or the final response to our study documents of prognosis with the confirmation of being alive prior to March 31, 2011; and (3) March 31, 2011, when the date of death, the final hospital visit, or the final response to our study documents of prognosis were later than April 1, 2011.

### Ethics

The ethical committee at the Cardiovascular Institute granted ethical permission for this study and all the patients gave written informed consent.

### Definitions

The present study included information on baseline characteristics of patients and their cardiac history, risk factors, and medications at initial PCI. An estimated glomerular filtration rate (GFR) was calculated by using the GFR equation used for the Japanese population; GFR = 194*(serum creatinine)^−1.094^*(age)^−0.287^*(0.739 if female) [7]. Chronic kidney disease (CKD) was defined as a baseline eGFR ≤60 ml/min/1.73 m^2^ [8]. BMI was calculated at initial PCI by dividing the patient’s measured weight (in kg) by the square of the height (in m). Obesity was defined as BMI ≥25 kg/m^2^ [9, 10]. Progression of a new lesion was defined as the development of a coronary lesion that was not significant at initial PCI but was requiring additional PCI due to ischemic symptoms and/or abnormal results of functional study including fractional flow reserve, radionuclide myocardial perfusion imaging using exercise or pharmacological stress, and treadmill exercise testing [11, 12]. We defined angiographic significant stenosis as >50 % diameter stenosis. Multi-vessel disease was defined as ≥2 vessel disease with angiographic significant stenosis.

### Statistical analysis

The Kaplan–Meier curve was used to assess the survival rates without new-lesion PCI. In the patients’ background, the categorical and continuous data are presented as number (%) and mean ± standard deviation, respectively. The Chi-square test was used for group comparison, and the unpaired Student’s *t* test was used for the comparison of continuous variables between the two groups. Univariate Cox regression analysis was used to identify the co-factors with significant effects on new-lesion PCI. Multivariate Cox regression analysis (step-wise method) including significant univariate variables and marginally significant variables (*p* < 0.10) in the univariate analysis was performed to determine the independent prognostic factors for new-lesion PCI. These analyses were performed using SPSS (SPSS Inc., Chicago, IL, USA) for Windows (Microsoft Corp., Redmond, WA, USA), version 14.0 software. Statistical significance was set at a two-sided *p* value of <0.05.

## Results

**Fig. 1:**
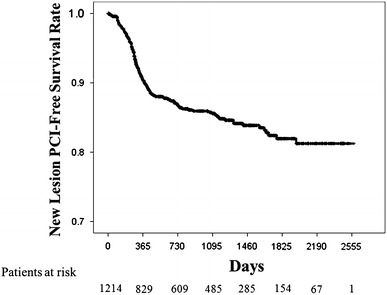
Kaplan–Meier curve for survival rates without new-lesion PCI. Degree of angiographic stenosis of right coronary artery (RCA) (**a**), left anterior descending coronary artery (LAD) (**b**), and left circumflex coronary artery (LCx) (**c**) at initial percutaneous coronary intervention (PCI) and follow-up coronary angiography (CAG) is shown

Progression of new coronary lesion was observed in 152/1,214 patients in this study. The median follow-up period was 1,032 ± 704 days. The cumulative rate of new-lesion PCI was 9.5 % at 1 year, 14.4 % at 3 years, and 17.6 % at 5 years (Fig. 1). There were no difference in age, sex, traditional coronary risk factors including hypertension, diabetes mellitus, dyslipidemia, cigarette smoking, family history of CAD, and CKD. Total cholesterol, low-density lipoprotein cholesterol, triglyceride level, fasting glucose, and HbA1c level at baseline PCI were comparable. Prevalence of MVD was more common in patients with new-lesion PCI (82.2 vs. 56.9 %, *p* < 0.001). High-density lipoprotein cholesterol (HDL) level at primary PCI was significantly lower in patients with new-lesion PCI than those without (47.0 ± 11.9 vs. 51.4 ± 15.1 mg/dl, *p* < 0.001). Prevalence of medications including dual anti-platelet therapy, anti-coagulation therapy, beta-blockers, angiotensin-converting enzyme inhibitor (ACE-I)s, angiotensin II receptor blocker (ARB)s, calcium-channel blockers, vasodilators, statins, and hypoglycemic drugs were comparable between the two groups. Patients using insulin (5.9 vs. 2.7 %, *p* = 0.035) were more common in patients with new-lesion PCI (Table 1).

**Table 1:** Patients’ background characteristics

	Non-PCI (*n* = 1,062)	Non-culprit lesion PCI (*n* = 152)	*p* value
Age (years)	65.4 ± 11.0	65.3 ± 9.9	0.858
Male	81.9 %	86.2 %	0.196
Hypertension	63.6 %	68.4 %	0.242
Diabetes mellitus	33.5 %	36.2 %	0.516
Dyslipidemia	59.6 %	59.2 %	0.926
Cigarette	36.3 %	40.1 %	0.366
Family history	15.8 %	16.4 %	0.843
CKD	31.9 %	34.2 %	0.572
Obesity	38.1 %	47.4 %	0.028
Past PCI	10.5 %	11.2 %	0.811
OMI	11.0 %	11.8 %	0.762
ACS	38.6 %	37.5 %	0.793
MVD	56.9 %	82.2 %	<0.001
LMT disease	5.6 %	8.6 %	0.143
Laboratory data at baseline PCI			
Total cholesterol (mg/dl)	192.2 ± 39.3	193.3 ± 35.8	0.743
LDL (mg/dl)	112.8 ± 33.6	116.3 ± 30.5	0.227
HDL (mg/dl)	51.4 ± 15.1	47.0 ± 11.9	<0.001
TG (mg/dl)	150.8 ± 111.6	155.5 ± 101.2	0.620
Glucose (mg/dl)	133.7 ± 54.2	138.3 ± 61.7	0.345
HbA1c (%)	6.1 ± 1.2	6.2 ± 1.4	0.498
Medications after baseline PCI			
DAPT	95.0 %	98.0 %	0.097
Anti-coagulant therapy	7.9 %	5.9 %	0.389
Beta-blockers	36.2 %	42.1 %	0.155
ACE-Is	15.9 %	17.8 %	0.562
ARBs	38.0 %	43.4 %	0.203
CCBs	44.4 %	44.7 %	0.929
Vasodilators	35.3 %	38.8 %	0.399
Statins	61.4 %	55.3 %	0.148
Hypoglycemic drug	19.1 %	23.0 %	0.256
Insulin	2.7 %	5.9 %	0.035

*CKD* chronic kidney disease, *Past PCI* past history of percutaneous coronary intervention, *OMI* history of myocardial infarction, *ACS* acute coronary syndrome, *MVD* multi-vessel disease, *LMT* left main trunk, *LDL* low-density lipoprotein cholesterol, *HDL* high-density lipoprotein cholesterol, *TG* triglyceride, *DAPT* dual anti-platelet therapy, *ACE-I* angiotensin-converting enzyme inhibitor, *ARB* angiotensin II receptor blocker, *CCBs* calcium channel blocker, *Statin* HMG-CoA inhibitor

Data are expressed as mean ± deviation, or counts (percentage)

Univariate Cox regression analysis showed that obesity (*p* = 0.041, hazard ratio 1.393, 95 % CI 1.013–1.915), MVD (*p* < 0.001, hazard ratio 3.249, 95 % CI 2.143–4.924), HDL (*p* < 0.001, hazard ratio 0.977, 95 % CI 0.965–0.990), ARB use (*p* = 0.048, hazard ratio 1.383, 95 % CI 1.003–1.906), and insulin use (*p* = 0.037, hazard ratio 2.048, 95 % CI 1.044–4.017) were associated with new-lesion PCI (Table 2). Multivariate Cox regression analysis (step-wise) including significant univariate factors as well as marginally significant ones (*p* < 0.10) (dual anti-platelet therapy and left main trunk) showed that MVD (*p* < 0.001, hazard ratio 2.921, 95 % CI 1.922–4.439), lower HDL (*p* = 0.003, hazard ratio 0.980, 95 % CI 0.967–0.993), and insulin use (*p* = 0.039, hazard ratio 2.050, 95 % CI 1.043–4.029) were the independent determinants of new-lesion PCI (Table 3).

**Table 2:** Unadjusted predictors for the development of new lesions

	*p* value	Hazard ratio	95 % CI
Age (years)	0.647	1.003	0.989–1.019
Male	0.197	1.355	0.855–2.148
Hypertension	0.129	1.304	0.926–1.835
Diabetes mellitus	0.394	1.155	0.829–1.607
Dyslipidemia	0.831	1.036	0.750–1.432
Cigarette	0.833	1.036	0.749–1.432
Family history	0.780	0.941	0.613–1.445
CKD	0.170	1.265	0.904–1.769
Obesity	0.041	1.393	1.013–1.915
Past PCI	0.472	1.203	0.727–1.993
OMI	0.982	1.108	0.678–1.813
ACS	0.567	0.909	0.654–1.262
MVD	<0.001	3.249	2.143–4.924
LMT disease	0.057	1.737	0.984–3.067
Total cholesterol	0.703	0.999	0.995–1.003
LDL	0.632	1.001	0.996–1.006
HDL	<0.001	0.977	0.965–0.990
TG	0.483	1.000	0.999–1.002
Glucose	0.147	1.002	0.999–1.005
HbA1c	0.405	1.055	0.930–1.196
DAPT	0.075	2.829	0.902–8.876
Anti-coagulant therapy	0.447	0.770	0.393–1.510
Beta-blockers	0.167	1.255	0.909–1.732
ACE-Is	0.986	0.996	0.657–1.511
ARBs	0.048	1.383	1.003–1.906
CCBs	0.804	1.041	0.756–1.434
Vasodilators	0.460	1.131	0.816–1.567
Statins	0.177	0.802	0.583–1.105
Hypoglycemic drugs	0.157	1.314	0.901–1.917
Insulin	0.037	2.048	1.044–4.017

*CKD* chronic kidney disease, *Past PCI* past history of percutaneous coronary intervention, *OMI* history of myocardial infarction, *ACS* acute coronary syndrome, *MVD* multi-vessel disease, *LMT* left main trunk, *LDL* low-density lipoprotein cholesterol, *HDL* high-density lipoprotein cholesterol, *TG* triglyceride, *DAPT* dual anti-platelet therapy, *ACE* angiotensin-converting enzyme inhibitor, *ARB* angiotensin II receptor blocker, *CCBs* calcium channel blocker, *Statin* HMG-CoA inhibitor, *CI* confidence interval

**Table 3:** Adjusted determinants of the development of new lesions

	Univariate *p* value	Hazard ratio	95 % CI	*p* value
MVD	<0.001	<0.001	2.921	1.922–4.439
HDL	<0.001	0.003	0.980	0.967–0.993
Insulin	0.037	0.037	2.050	1.043–4.029

*MVD* multi-vessel disease, *HDL* high-density lipoprotein, *CI* confidence interval

## Discussion

The major findings of the present study are the following. The cumulative rate of new-lesion PCI was 9.5 % at 1 year, 14.4 % at 3 years, and 17.6 % at 5 years. New-lesion PCI continued to occur beyond 1 year after PCI without attenuation of their annual incidences up to 5 years. Low HDL, MVD, and insulin use at primary PCI were the independent predictors for the incidence of long-term new-lesion PCI in Japanese CAD patients who underwent PCI.

New coronary lesion PCI was observed in 152/1,214 of patients who underwent primary PCI in this study. The cumulative rate of new-lesion PCI was 9.5 % at 1 year, 14.4 % at 3 years, and 17.6 % at 5 years. A recent study showed that approximately 6 % of patients who underwent PCI have clinical plaque progression requiring additional PCI by 1 year [12], therefore, the incidence of new-lesion PCI at 1 year after primary PCI seemed to be relatively high. A higher rate of routine follow-up CAG in Japanese clinical practice might be attributed to the higher incidence of new-lesion PCI than in Western countries. Hence, the incidence was similar to previous reports with Japanese large registry [13]. Although, the clinical efficacy of routine follow-up CAG after PCI was not established, the indication of PCI was determined by not only angiographic findings but also the evidence of ischemia, which includes patient’s symptom and/or abnormal results of functional study at our institute. Thus, ischemic coronary lesions either symptomatic or asymptomatic was developed more frequently than expected.

There is a well-known inverse relationship between the level of HDL and the presence and the development of coronary artery disease [14]. The protective effect of HDL on atherosclerosis is suggested by previous studies that higher HDL was associated with longevity and freedom from coronary artery disease [15, 16]. Experimental studies demonstrated that HDL had various anti-atherosclerotic effects through mediating macrophage cholesterol efflux [17], maintaining endothelial function [18, 19], protecting against oxidation of LDL [20], attenuating inflammation [21], and interfering with thrombotic component [22–25]. Despite the established evidence of an inverse relationship between the level of HDL and the cardiovascular risk, the optimal medical treatment for the patients with low HDL has not been firmly established. ATP III [26] recommends the following approach to the patients with low HDL such as intensify weight management, increase physical activity, smoking cessation, and medical therapy including statin, fibrate, and ezetimibe, etc. However, the current medical treatment is not enough for the patients with low HDL, and further development in this area is awaited. Interestingly, high LDL was not a predictor of new lesion progression in the present study. As well as low HDL, high LDL is an established risk factor of atherosclerotic diseases. However, in current clinical practice, physicians routinely administered statin treatment for patients with high LDL who underwent PCI as a secondary prevention therapy in current clinical practice, and statins were quite effective in most of the cases. These matters might contribute to the attenuation of the impact of high LDL level on new lesion progression in the present study.

MVD was also an independent predictor of the progression of new coronary lesions. In agreement with the present study, Park et al. [11] previously reported that an increased number of significant coronary artery lesions was associated with clinically driven PCI for new lesions after primary PCI. Glaser also reported that patients with multi-vessel CAD during original PCI were more likely to require non-target lesion PCI, suggesting that greater CAD burden conferred a significantly higher risk for clinical plaque progression [12]. Severity of coronary artery atherosclerosis at primary PCI was understandably associated with future development of atherosclerotic lesions.

Insulin-treated DM was an established risk for atherosclerosis [27–29]. In the present study, insulin use at primary PCI was the independent predictor of the long-term incidence of new-lesion PCI. Several possible explanations are suggested for this phenomenon. First, insulin use is strongly associated with more advanced and severe DM. In addition, various potentially harmful effects of insulin have been identified. Weight gain is another problem in patients with insulin-dependent DM. Furthermore, insulin has been shown to be associated with endothelial dysfunction [30] and it is known that patients treated with insulin often have reduced responsiveness to antiplatelet treatment [31].

The present study showed that MVD, lower HDL, and insulin use were independent predictors of the development of new coronary artery atherosclerosis after PCI. Risk stratification of patients with CAD after PCI by the presence of these risk factors might be useful in clinical practice. On the other hand, we could not show how to prevent such progression of coronary atherosclerosis in patients at high risk. Further study will be needed for elucidating the optimal medical therapy for high-risk patients with CAD after PCI.

We recognize that the present study has several limitations. This study was based on a single center’s experience and the results of this study cannot be generalized to all medical centers. Because of the limited sample size, the statistical power might not be strong enough for any negative data such as other lipid profiles and the presence of the metabolic syndrome to be conclusive.

## Conclusions

Progression of new coronary lesions was common and new-lesion PCI continued to occur beyond 1 year after PCI without attenuation of their annual incidences up to 5 years. MVD, lower HDL, and insulin use were the independent predictors of the progression of new coronary lesions in Japanese CAD patients who underwent PCI.
